# Annotations

**Published:** 1900-11-03

**Authors:** 


### ANNOTATIONS.

### Typhoid and the Tea-kettle.

Again we are sadly reminded of this curse of armies, enteric fever, and are led to ask how far the lessons of the beginning of the year have been applied to prevent a recurrence of that prevalence of fever which has been the one great and real failure of the war. England for so long stood before the nations as the leader in sanitary affairs that the grief felt for the loss of so many of her sons from typhoid fever has been intensified by the humiliation at having to confess that, much as we have shown ourselves able to do in protecting a stationary population from the ravages of this disease, we have been thoroughly beaten in our efforts to shield our moving troops from its attacks. The matter, however, was condoned by our military successes. That it had been found essential to run certain risks was generally admitted, and that these risks had brought their penalty was accepted as a military necessity. But with the reopening of railways, the establishment of government, and the resumption of trade there still remains an ominous prevalence of typhoid fever. We may take it that these cases which we hear of day by day, this sad death in our Royal Family, these sufferers from enteric among the C.I.V.'s on board the *Aurania*, all became infected within the last five or six weeks, showing how persistent is the germ, and how great the danger, and unfortunately suggesting that we have not yet learned the art of preventing this so-called preventable disease. There is much that is speculative in regard to typhoid fever and its relation to drinking water. But there is one thing that is known, namely, that water which is freshly boiled is innocent of infection. At the present time people in South Africa are very anxious as to the coming summer and the likelihood of fever, and there is much questioning as to the water supply. From Cape Town to Pretoria the railway route is thoroughly polluted; and although in a settled country like England we can trust to purification of our water supply on the large scale, experience has shown that, as an extemporary means, there is only one way to safety in this respect, and that is by drinking nothing except what has been freshly boiled. This "policy of the tea-kettle," as the late Ernest Hart used to call it—the policy, that is, not of great regimental boilers, but of small kettles by which each small group of men can ensure that whatever they drink has been recently boiled (sterilised, as the phrase now goes)—is the only known protection against the spread of typhoid fever by means of water, where the water is open to suspicion, as it is all over South Africa; and it may well be asked—now that the railways are open and transport is available—how far the troops are supplied with tea-kettles and petroleum stoves by which to ward off typhoid fever. The frequency with which we hear of this disease makes us question whether even yet our troops enjoy the full advantage of modern knowledge on the subject.

### Filth and Fashion.

Much as we hesitate to encroach upon æsthetic problems especially when they are so abstruse and so complicated as those must be which touch upon the attractiveness of woman, we feel drawn to join in the protest against the fashion of the trailing skirt. Without going to such an extreme as to say that man is the object aimed at by woman's dress, there would seem to be much probability in the idea that one object at least of feminine attire is to add to feminine attractiveness, and if we analyse the sensations produced by dress, and regard one by one the component elements which go to make a well-dressed woman pleasant to the eye, and even seductive—if one likes the phrase—one sees that each item in her costume must indicate something or raise some idea about the woman herself. The furs and velvets on an icy day bring up before one's mind's eye warmth and comfort; the well-gloved hand and the neat chaussure suggest a graceful and well-proportioned frame; the little frills and bits of lace peeping out beneath the skirts or round the neck, the whiff of lavender as she passes, and even the "rustle" as she moves all raise visions of that neatness, that sweetness, and that cleanliness in every particular which should form part of our ideal of womanhood.

Some boisterous hoydens laugh at frills and furbelows, but they have their meaning, and to rough-and-tumble man, fresh from the dirt and grime of the outer world, all these little things suggest just that contrast with his own life which makes him put woman on a pedestal. Even the trailing skirt when it sweeps a rich carpet in a cleanly home may carry still further the same idea, hinting as it does at purity of surroundings. In everything except the mere clothing of the body woman's dress suggests the nature of the woman, and is beautiful in proportion as it suggests what we admire.

But what about the trailing skirt that sweeps the streets, the skirt that drags in dust and mire, the skirt that licks up dirt unmentionable and carries all these abominations into the lady's dwelling? What about the suggestiveness of dress when dress does this? Seriously, it is a matter of much importance. If there is any truth in modern theories as to the causation of disease, women do a grave injury to those with whom they live by this dirty habit of dragging into their houses the rubbish of the streets. But on the æsthetic grounds—which we take it are the sole grounds except the mere question of warmth which woman considers in regard to her costume—surely the trailing skirt worn out of doors is utterly wrong. The dress should and does suggest the woman, and the woman suggested by the muddy skirt is not a nice one.

### Tracing the Doctor.

A question of considerable importance has lately been raised in regard to the propriety of medical men who dispense their own medicine having their names and addresses printed on the labels of their physic bottles.

There is no doubt that a very large number of most respectable medical men—many of the best, indeed—would regard it as *infra dignitate* to use any but a plain label, holding that anything in the nature of name or address or notification of hours of consultation—anything, in fact, beyond the mere direction how the medicine is to be taken savours of advertisement, and is to be looked upon with extreme disfavour.

There are certain practical advantages, however, in being able to trace the doctor from whom medicine has been obtained, and in regard to medicines containing poisons it seems a question whether it is not obligatory upon the medical man supplying such medicines to affix his name and address upon each bottle. According to the *British Medical Journal* there seems to be good reason, on public grounds, for requiring that whoever dispenses medicines containing poisons shall place his name and address upon the bottle. The legal interpretation of Section III. of the Pharmacy Act Amendment Act 1869 appears to be that this section relieves qualified medical practitioners when supplying their patients with medicines containing poisons from the penalties of Section XVII. of the 1868 Act, provided that such medicines are labelled with the name and address of the medical man. This means, apparently, that there is an obligation upon medical men dispensing such medicines to label them in this way. Certainly such a proceeding is in accordance with common sense, and, indeed, we would go further and say that even medical men who write prescriptions ordering poisons ought to sign their prescription in such a manner that they can be traced, so that in case of accident or error inquiries can be made without loss of time. One of the curious by-laws of the Royal College of Physicians lays it down that every Fellow or Member of the College in prescribing for a patient shall sign "the initial letters of his name." Fortunately most physicians have the good sense to print their address upon their notepaper, which partly gets over the difficulty; but there can be no doubt that it would be far better if it were made compulsory upon prescribers as well as dispensers to put their names honestly to their work, so as to prevent all difficulty in tracing the doctor.

### How to People Africa.

Now that so much of South Africa has been added to the British Empire, it is desirable that peacefully, but surely, British ideas should spread there. This can only be done by leavening the whole population with a large and wholesome British element. There will, no doubt, be a large influx of English to the towns, but that will hardly meet the case. All our colonies suffer from the entrance into their midst of men who are fitted only for town life; and in countries where there is accommodation for a much larger population than exists, there is yet overcrowding, misery, and competition in the towns. What is needed is people who will settle in the country. This is pointed out in a letter which the Duke of Argyll contributed recently to the *Times*. The Duke points out that "our chief difficulty will be to get the country districts leavened with Britishers." The Dutch language, the Dutch idea, and therewith the Dutch discontent with British rule will linger in the country districts unless peaceful neighbours of British descent mingle with the Boers. To this end the Duke suggests that selected boys from our industrial schools should be sent to South Africa, and either placed singly on farms, or formed into colonies under proper supervision, and taught the industries of country life. Many of the boys in our industrial schools are not really bad boys; they are only boys whose energies have never had a wholesome field for their exercise, and it is quite possible that the "hooligan" of the slums might become a good citizen in a place where he had wholesome occupation for his superabundant vitality. Might not Dr. Barnardo, and the heads of other institutions which reclaim the waifs from our streets and export them to Canada, send a few to South Africa? Before long there will be a good enough demand for healthy youngsters who have the will and the strength to work. Major-General Hector Macdonald also has his suggestion. He desires colonists from his own district, the Black Isle. And it is almost certain that any who accept his invitation and venture to go to Africa will find that life on the veldt is at least not harder nor worse remunerated than life on a croft in the Scottish Highlands.

### Rats and Plague.

As yet another illustration of the relation between plague in rats and in human beings, and of the extreme probability that it is through the rats that the disease is spread, reference may be made to the official report by Professor Kitasato of Tokio on recent outbreaks of plague at Kobé and Osaka, summarised for presentation to the Académie de Médecine of Paris by M. Vallin.

From this it appears that in the town of Kobé, which has a population of 230,000 inhabitants, there were 23 cases of plague between November 3rd and December 21st, 1899, while in Osaka, with 750,000 inhabitants, there were 39 cases of plague from November 18th, 1899, to January 11th, 1900.

If, however, we turn to the rats we see how much more prevalent the disease was among them than among the humans. The municipality of each of these towns paid a reward for every rat dead or alive, and from the middle of November to the end of January 20,000 rats were taken at Kobé, of which 20 per cent. were infected, and 15,000 at Osaka, of which 10 per cent. were infected. Thus even of the rats collected—probably a very small portion of the whole number attacked—4,000 were shown to be infected in Kobé and 1,500 in Osaka, against 23 and 39 cases respectively among human beings.

That plague can occur and spread in the absence of rats is obvious enough; but it seems to have been shown in many instances besides the above that it may exist primarily as a rat epidemic by which these creatures may be decimated, while only a few human beings are attacked. So long, however, as the rats are infected man is never safe, and in all cases the destruction of rats should be insisted on.

### A Question of "Consent."

According to the New York *Medical Record*, a physician in New Bedford, United States, in the course of his duties as what we should call public vaccinator, visited a house in which he found a six-year-old boy who had not been vaccinated. He thereupon vaccinated the lad without any objection being raised. But there was also a baby, and in regard to the vaccination of the infant objections were raised by the mother. To these, however, the doctor answered that she must settle all that with the Board of Health, and he proceeded to perform the operation. Some time later he found himself dragged into court on a charge of assault and battery for so doing. Now here comes the interesting point. It was proved that the mother, notwithstanding her remonstrances, had bared the infant's arm for the vaccination, and the court acquitted the defendant, holding that what the mother had done to facilitate the proceeding showed that, although undoubtedly reluctantly, she had in the end consented.

